# By wind or wing: pollination syndromes and alternate bearing in horticultural systems

**DOI:** 10.1098/rstb.2020.0371

**Published:** 2021-12-06

**Authors:** Gabriela Garcia, Bridget Re, Colin Orians, Elizabeth Crone

**Affiliations:** Department of Biology, Tufts University, Medford MA 02155 USA

**Keywords:** alternate bearing, crop yields, pollination syndrome, fruit, nuts, economies of scale

## Abstract

Cyclical fluctuations in reproductive output are widespread among perennial plants, from multi-year masting cycles in forest trees to alternate bearing in horticultural crops. In natural systems, ecological drivers such as climate and pollen limitation can result in synchrony among plants. Agricultural practices are generally assumed to outweigh ecological drivers that might synchronize alternate-bearing individuals, but this assumption has not been rigorously assessed and little is known about the role of pollen limitation as a driver of synchrony in alternate-bearing crops. We tested whether alternate-bearing perennial crops show signs of alternate bearing at a national scale and whether the magnitude of national-scale alternate bearing differs across pollination syndromes. We analysed the Food and Agriculture Organization of the United Nations time series (1961–2018) of national crop yields across the top-producing countries of 27 alternate-bearing taxa, 6 wind-pollinated and 21 insect-pollinated. Alternate bearing was common in these national data and more pronounced in wind-pollinated taxa, which exhibited a more negative lag-1 autocorrelation and a higher coefficient of variation (CV). We highlight the mutual benefits of integrating ecological theory and agricultural data for (i) advancing our understanding of perennial plant reproduction across time, space and taxa, and (ii) promoting stable farmer livelihoods and global food supply.

This article is part of the theme issue ‘The ecology and evolution of synchronized seed production in plants’.

## 1. Introduction

Variation in plant reproduction is central to processes from forest dynamics to farmer livelihoods [1,2]. In perennial plants, masting (synchronous, highly variable reproduction) marks one extreme end of the spectrum of population-level variation, and constant yield marks the opposite end. To date, much of the research on synchronous seed production has been focused on mast-seeding by wind-pollinated trees in temperate regions [3,4]. It may be that mast-seeding is more common in wind-pollinated taxa; theory suggests selection for enhanced pollination efficiency through synchronous flowering with conspecifics is more likely in wind-pollinated species [5,6]. In insect-pollinated species, synchronous flowering may saturate insect pollinators and high pollination efficiency at low flowering density may select for a more constant production of flowers ([7], but see [8,9]).

**Figure 1.**
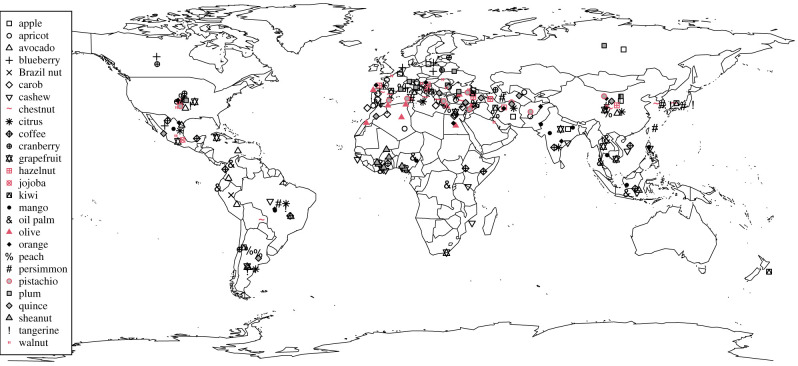
Spatial distribution of the top-producing countries for each alternate-bearing crop. Red symbols are wind-pollinated and black are insect-pollinated.

An alternative explanation of the overrepresentation of wind-pollinated species in the synchronous seeding literature is that much of the masting work, and indeed the bulk of ecological and evolutionary research, has been done in temperate regions where wind is the predominant pollination syndrome among forest trees [4,10–12]. Early reviews on whether pollination syndrome predicts the tendency for masting had difficulty gathering sufficient data on insect-pollinated and animal-dispersed taxa [5,6]. A recent meta-analysis [13] included data with approximately equal numbers of animal- and wind-pollinated species, but there were more time series per species for the wind- than animal-pollinated ones (Ian Pearse 2021, personal communication). To help to fill this gap, we make use of an analogous pattern of highly variable reproduction in perennial crop plants which, unlike mast-seeding forest trees, are biased toward insect-pollinated taxa and span tropical, Mediterranean and temperate climates (figure 1). Alternate (or biennial) bearing in fruit and nut crops is an intermediate pattern of perennial reproductive variability in which a year of high reproduction is followed by a year of low reproduction [2,14,15]. While media and trade reports have cited alternate bearing in discussions of national crop yield [16,17], literature on the extent and drivers of synchrony among alternate-bearing individuals is scarce.

Despite evidence of similar plant-level mechanisms in masting and alternate bearing [4,18–21], ecological research on the synchrony of mast-seeding has largely ignored, or explicitly excluded, alternate-bearing crops ([13]; though notable exceptions include work on citrus and pistachio [22–24]). This may be because breeding and management actions are generally assumed to outweigh any natural conditions that could result in alternate bearing at farm-, region- or nationwide scales [2,13,25,26]. Similarly, agricultural research on yield and alternate bearing rarely integrates insights from masting literature. Such insights include the possibility that factors which increase yield in one year may result in a more severe reduction in the following year [27] and the expectation that wind-pollinated taxa may be more prone to synchronous fluctuations in yield at larger spatial scales than insect-pollinated taxa [6,13,28]. Here, we use global crop production data for plants known to be alternate bearing at an individual level [2,14] to evaluate patterns of seed production at the national level. Specifically, we assess (i) whether these crops are alternate bearing at national scales and (ii) whether patterns differ across pollination syndromes and are consistent with findings in masting systems [5,6,13].

For this analysis, we use data from the Food and Agriculture Organization of the United Nations (FAO) [29]. The global, long-term nature of the FAO data offers a unique opportunity to study reproductive patterns in perennial plants, but also poses some limitations that could mask a signal of alternate bearing even if one exists. First, the FAO reports data at the national level, and while the total area under production is included, it is not spatially explicit (i.e. we do not know if it is contiguous or scattered across a large geographical area) and thus cannot serve as a proxy for the extent of cultivation. As such, we cannot directly test for synchrony among individuals or populations using these data. While a signal of alternate bearing in national-scale data would require synchrony at smaller scales, a lack of a signal does not preclude synchrony at the farm or regional level. Studies of synchrony in mast-seeding species suggest that we might expect signals of alternate bearing to be weaker in data at national compared to local scales [30]. In that sense, our analysis is likely to underestimate the magnitude of alternate bearing at more local scales and thus their potential impact on farmer livelihoods. Furthermore, multiple crops are sometimes grouped together into a single FAO crop category (such as lemons and limes), which could mask a species-specific signal [31]. Finally, there is no information on the genetic variety or cultivation practices employed in each country that could influence the tendency toward alternate bearing within taxa [32]. These features make any observed patterns in these data particularly salient. The presence of alternate bearing at national scales would highlight its ecological and socio-ecological importance [14,33]. A national-scale analysis also allows for broad comparison with masting species where such synchrony has been observed [34,35].

In this study, we characterized alternate bearing at national scales using three complementary metrics: lag-1 autocorrelation (the tendency for high-seed years to be followed by low-seed years), coefficient of variation (CV) in seed production (how variable seed production is at national scales) and bimodality of seed production (whether high and low years are more common than average years). Classic alternate bearing would have all three metrics at a population level; in our study, we test whether they are detectable at a national scale. Throughout, when we discuss alternate bearing at a national scale, we refer to any or all of these variables. We had strong *a priori* expectations that wind-pollinated species would show stronger alternate bearing at national scales, i.e. they would have stronger negative lag-1 autocorrelation, higher CV and possibly stronger bimodality than insect-pollinated species. After assessing these patterns at national scales, we discuss the socio-ecological implications of our findings and explore future research directions.

## 2. Methods

### (a) Data selection, modification and validation

We downloaded 58 years (1961–2018) of data collected by the FAO on yield and production for 27 perennial crop taxa that have been reported in the horticultural literature as having a tendency toward alternate bearing at the individual level [2,14]. For each crop, we selected up to 10 countries worldwide, each with at least 20 years of data, for inclusion in the analysis by their highest total production. The included nations were filtered for historical consistency and the crop names were modified for interpretability (table 1; details in electronic supplementary material S1). The resulting dataset was comprised of 236 crop–country combinations (figure 1). We performed the country selection and all subsequent analyses twice, once including only the past 25 years (1994–2018, a period during which reporting has been more consistent) and once including all data on record (back to 1961 for some crops and countries). Patterns did not differ between the two time series; the results and discussion below describe the ‘long’ time series (please refer to electronic supplementary material S2 for results of the ‘short’ time series).

**Table 1.** Data description. FAO data are at national scales and the time coverage spans from 1961 to 2018. Latin names were sourced from the FAO commodity definitions. Pollination syndrome was determined through a search of the available literature.

FAO crop name	short name	Latin name(s)	pollination syndrome	countries: no. (max. years)
apples	apple	*Malus pumila; M. sylvestris; M. communis; Pyrus malus*	insect	10 (58)
apricots	apricot	*Prunus armeniaca*	insect	9 (58)
avocados	avocado	*Persea americana*	insect	9 (58)
blueberries	blueberry	*Vaccinium myrtillus; V. corymbosum*	insect	8 (58)
Brazil nuts, with shell	Brazil nut	*Bertholletia excelsa*	insect	1 (58)
carobs	carob	*Ceratonia siliqua*	insect	10 (58)
cashew nuts, with shell	cashew	*Anacardium occidentale*	insect	10 (58)
chestnut	chestnut	*Castanea vesca; C. vulgaris; C. sativa*	wind	10 (58)
coffee, green	coffee	*Coffea arabica; C. robusta; C. liberica*	insect	10 (58)
cranberries	cranberry	*Vaccinium macrocarpon; V. oxycoccus*	insect	10 (58)
grapefruit (inc. pomelos)	grapefruit	*Citrus maxima; C. grandis; C. paradisi*	insect	7 (58)
hazelnuts, with shell	hazelnut	*Corylus avellana*	wind	10 (58)
jojoba seed	jojoba	*Simmondsia chinensis*	wind	9 (58)
karite nuts (sheanuts)	sheanut	*Butyrospermum parkii*	insect	1 (35)
kiwi fruit	kiwi	*Actinidia chinensis*	insect	9 (47)
lemons and limes	citrus	*Citrus limon; C. aurantifolia; C. limetta*	insect	10 (58)
mangoes, mangosteens, guavas	mango	*Mangifera indica*	insect	10 (58)
oil palm fruit	oil palm	*Elaeis guineensis*	insect	10 (58)
olives	olive	*Olea europaea*	wind	10 (58)
oranges	orange	*Citrus sinensis; C. aurantium*	insect	10 (58)
peaches and nectarines	peach	*Prunus persica; Amygdalus persica; Persica laevis*	insect	9 (58)
persimmons	persimmon	*Diospyros kaki; D. virginiana*	insect	9 (58)
pistachios	pistachio	*Pistacia vera*	wind	9 (58)
plums and sloes	plum	*Prunus domestica; P. spinosa*	insect	10 (58)
quinces	quince	*Cydonia oblonga; C. vulgaris; C. japonica*	insect	7 (58)
tangerines, mandarins, clementines, satsumas	tangerine	*Citrus reticulata*	insect	10 (58)
walnuts, with shell	walnut	*Jugland regia*	wind	9 (58)

To confirm that the FAO dataset reflected historical reproductive output of agricultural perennials, we scanned horticultural and economic online publications on focal crops that mentioned environmental shocks that occurred in exceptionally low yielding years at national scales and looked for signals of these in the detrended FAO time series (see §2b below). This validation exercise was done as a qualitative check of how well these aggregate time series represent on-the-ground experiences of growers; therefore, we scanned the available online information, rather than attempting a systematic review of trade publications.

### (b) Time series analysis

All statistical analyses were performed in R Studio Software 3.5.0 [36]. To focus on interannual variation in yield, rather than broader patterns of increasing or decreasing yield, we detrended all time series using generalized additive models (GAMs) fit to time series of yield (production × area^−1^) through time. These were calculated with defaults from the *mcgv* package (v. 1.8–33; [37]) and appeared to provide reasonable fits to the data based on visual inspection (electronic supplementary material S3 contains the full set of raw and GAM-detrended crop-country time series). To confirm that the results were not dependent on the detrending method, we repeated the analysis using locally estimated scatterplot smoothing (LOESS) and differencing as alternative detrending methods. The observed patterns persisted; we report the GAM-detrended results below (please refer to electronic supplementary material S2 for the results using LOESS and differencing). While national production (tonnes) was used for selecting the top-producing countries included in the analysis, yield (hg × ha^−1^) was used for time series analysis because annual yield estimates should be less sensitive than production to additional variability from factors like farm expansion or contraction that are irrelevant to the present study.

We characterized each time series with three common mast-seeding metrics that provide complementary information about alternate bearing. The lag-1 autocorrelation (AC-1; literally, the correlation between points separated by 1 year) is a measure of alternate bearing that captures the tendency of high years to follow low ones [38]. We also inspected the autocorrelation function at lags up to 17 years (for 51-year time series, or 1/3 of the time series length for shorter time series) as a descriptive tool to understand patterns of yield that differ from alternate bearing. Our second metric, the CV, is typically the standard deviation divided by the mean. However, because the mean of a detrended time series is 0, we used the standard deviation of the detrended time series divided by the mean of the raw time series, a technique commonly applied in climate modelling [39,40]. Third, we used Hartigans' dip statistic (D) to test for bimodality. D is a measure of deviations from a unimodal distribution of values; significant differences would indicate that yield is multimodal (*sensu* [28]). D was rarely significant (see §3), so we did not perform further tests to distinguish bimodality from other multimodal distributions.

We analysed whether each masting metric differed as a function of pollination syndrome (wind versus insect; an explanation of pollination syndrome classification for each crop can be found in electronic supplementary material S4). We treat pollination syndrome as a binary variable as in previous masting studies [5,6], though relative dependence on insect pollinators may vary in more nuanced ways (such as via self-compatibility and parthenocarpy, [41,42]). Of the 27 crops, 6 were wind-pollinated and 21 were insect-pollinated (table 1). Because alternate bearing could be driven in part by phylogenetic relatedness, we conducted these tests using phylogenetically corrected linear mixed models (LMMs) with the trait as the predictor, the masting metric as the response, and random effects of plant phylogeny and country using the *lme4* package (v. 1.1.-26; [43]). The plant phylogeny was constructed using the function ‘phylo.maker’ in the R package *V.PhyloMaker* [44] using the GBOTB.extended mega-phylogeny (74 531 tips) as backbone and the default option ‘scenario 3’, in which the tip for a new genus is bound to the 1/2 point between the family root node and basal node. The resulting phylogeny had 35 tips because six of the crop categories (plums and sloes; lemons and limes; mangos, mangosteens, guavas; oranges; walnuts; persimmons) contained multiple species [31]. The phylogenetically corrected LMMs were run using the ‘phylo_lmm’ function (*lme4*) on all possible combinations of FAO crop species of these six categories (*n* = 216). Because the results were consistent across all iterations and varied minimally (*p*-values always below *p* < 0.01 for AC-1 and CV; always above *p* > 0.13 for the dip statistic), the mean Chi-square and *p*-values are reported below (a table of the results across all 216 iterations can be found in the electronic supplementary material S5). The only observed differences across iterations were driven by species in the ‘mango, mangosteen and guava’ category, which are grouped by the FAO despite distant phylogenetic relatedness. We also performed a phylogenetically corrected LMM to compare the relationship between AC-1 and CV [13,45].

## 3. Results

**Figure 2.**
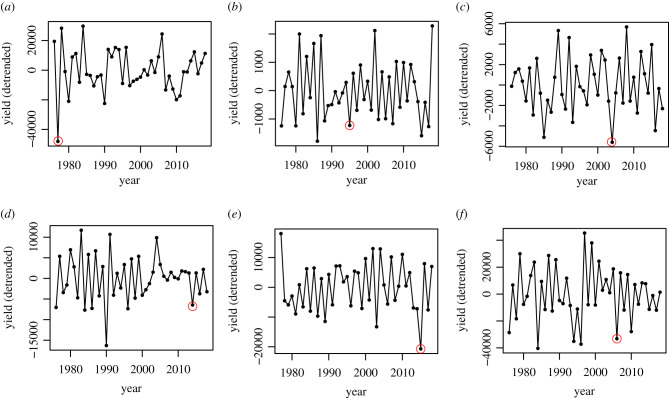
GAM-detrended time series of selected alternate-bearing crops. Data are at national scales from the FAO. Red circles indicate yield declines that coincide with published reports of problematic environmental conditions. (*a*) Apricot, Spain; (*b*) coffee, Brazil; (*c*) hazelnut, Turkey; (*d*) olive, Italy; (*e*) pistachio, United States; and (*f*) tangerine, Japan.

The validation exercise confirmed that the FAO time series were well-aligned with published reports on national shocks to production across pollination syndrome and countries (figure 2). Reports of poor weather and consequent pest outbreaks coincided with a dip in olive production in Italy in 2014 [17], and a warm winter in 2014 aligned with a drop in U.S. pistachio production in 2015 [46]. Drought is tied to the drop in Brazilian coffee production in 1995 [47]. Exceptionally cold weather was responsible for yield dips in Japanese tangerines in 2006 [48], Spanish apricots in 1977 [16] and Turkish hazelnuts in 2004 [49].

**Figure 3.**
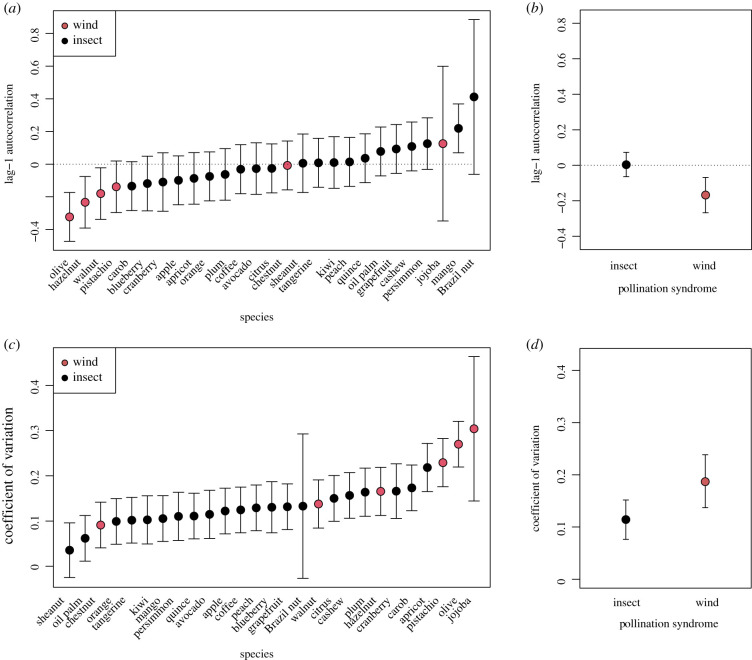
(*a*) Mean lag-1 autocorrelation values and 95% confidence intervals across alternate-bearing crops generated from the GAM-detrended FAO time series. (*b*) Among the crops in (*a*), those that are wind-pollinated have a significantly stronger lag-1 autocorrelation than those that are insect-pollinated. (*c*) Mean CV and 95% confidence intervals across alternate-bearing crops generated from the GAM-detrended FAO time series. (*d*) Those that are wind-pollinated have a significantly higher CV than those that are insect-pollinated.

Several crops exhibited alternate bearing at a national scale as indicated by a negative lag-1 autocorrelation (figure 3*a*). As predicted, the AC-1 was significantly more negative in wind-pollinated crops (−0.168; s.e. = 0.051) than insect-pollinated crops (0.003; s.e. = 0.035; *χ*^2^ = 11.55, d.f. = 1, *p* < 0.001; figure 3*b*). Similarly, the CV for wind-pollinated crops (0.187; s.e. = 0.03) was greater than their insect-pollinated counterparts (0.114; s.e. = 0.02; *χ*^2^ = 8.8, d.f. = 1, *p* = 0.003; figure 3*d*), supporting our *a priori* hypothesis that wind-pollinated species would show stronger alternate bearing. We did not find evidence of bimodality (only 1 out of 236 crop-country combinations was statistically significant at *p* < 0.05) and there was no significant effect of pollination syndrome (*χ*^2^ = 2.02, d.f. = 1, *p* = 0.15).

**Figure 4.**
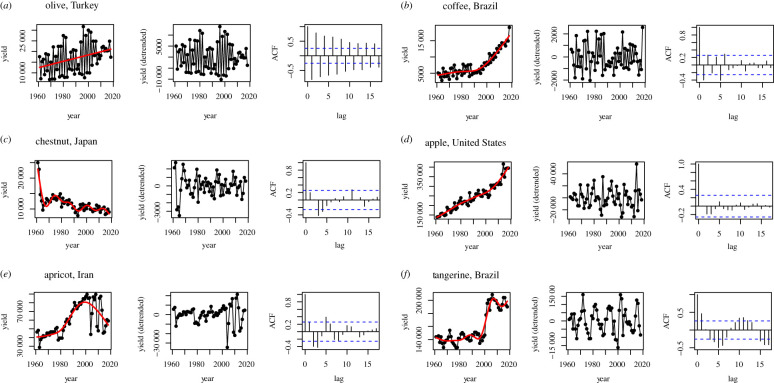
Examples of raw yield time series, detrended time series and autocorrelation plots for crops that exhibit strong alternate bearing (*a*,*b*), no alternate bearing (*c*,*d*) and longer reproduction cycles (*e*,*f*). ACF indicates ‘autocorrelation function’, i.e. the correlation between yield values in each time series and time intervals specified by the ‘lag’ on the *x*-axis.

Despite a general tendency towards stronger alternate bearing in wind-pollinated crops, there are interesting exceptions (figure 4; see also electronic supplementary material S3). For instance, some insect-pollinated crops (such as coffee in Brazil) show strong alternate bearing (figure 4*b*). The plotted crop by country values of AC-1 and CV also exhibit substantial scatter (electronic supplementary material S6), indicating that even within a crop type, the degree of alternate bearing at a national scale varies. Across all crops and countries, there was a negative correlation between AC-1 and CV (Pearson's *r* = −0.27, d.f. = 234, *p* < 0.001). This relationship was statistically significant after accounting for crop phylogeny (LMM of AC-1 versus CV: *χ*^2^ = 12.36, d.f. = 1, *p* < 0.001; slope ± s.e. = −0.62 ± 0.177).

Interestingly, some crops seemed to show cycles at longer scales, which was not an *a priori* expectation (figure 4*e*,*f*). As long cycles could be an artefact of the detrending method, we inspected their occurrence across each (GAM, LOESS and differencing) by counting the crop–country combinations that exhibited a significant positive lag followed by a significant negative lag and then returned to a positive lag (or vice-versa) in which the lag was greater than 1 year (i.e. excluding alternate bearing, which we accounted for in our primary analysis). Of the 236 crop–country combinations, GAM resulted in a total of 38, LOESS in 41, and differencing in 3 long cycles, with 2 cycles consistent across all detrending methods and 18 cycles present in both LOESS and GAM (see electronic supplementary material S7, for details).

## 4. Discussion

Alternate bearing is common among managed perennial crops at the national level. A negative lag-1 autocorrelation is significantly more common in wind- than insect-pollinated plants. Yield variability (CV) is also significantly greater in wind- than insect-pollinated crops. These results are in line with previous findings in natural plant systems that masting is more common and more pronounced among wind-pollinated taxa [5,6]. Bimodality was rare in these time series and did not differ between pollination syndromes, which is also consistent with natural systems where strong bimodality is uncommon and ‘partial masting’ prevails [3]. Given that we observed a strong signal of alternate bearing despite limitations of the FAO dataset (see §1), our findings offer encouraging support that enhanced integration of the alternate-bearing and masting literature could offer mutual insight.

Unsurprisingly, the CV of alternate-bearing crops reported here at national scales is smaller than has been observed for both masting and alternate-bearing taxa at population scales [6,35]. In general, alternate bearing leads to lower CVs than mast-seeding at longer intervals [38,50]. More importantly, we expect that in crop plants, as in wild plants, synchrony should decay with distance, and, at the present time, the scale of synchrony in crop plants is largely unknown. To explore this spatial scale somewhat quantitatively, we compared our results to data presented by Noble *et al*. [23], one of the few published studies of yield variability at multiple scales in a crop plant. Noble *et al*. [23] provide data on pistachio yield in four Californian counties. In their data, farm-level yield (CV 1.10) was more variable than county-level yield (CVs ranging from 0.47–0.67; see the differenced time series in their electronic supplementary material data S2 and S3). These are all higher than estimates of pistachio CV at a national scale from the FAO data presented here (0.34 for USA, range 0.05–0.43 across top 10 producing countries). This very limited exploration suggests that synchrony across crop yields decays with distance, and that further exploration of farm- to regional-scale data for crop plants could be valuable for understanding spatial synchrony, especially in insect-pollinated species.

Yield stability is frequently the goal of farmers and horticultural researchers [15,33], yet our results suggest that alternate bearing persists even at a national level. At the present time, studies on drivers of synchrony in agricultural settings are scarce and would benefit from integration with ecological theories about the causes of mast-seeding. For example, numerous studies of crop pollination are based on the premise of enhancing yield in a single year [51–53]. However, if the alternate bearing is due to resource depletion after high-seed years, then maximizing yield in 1 year could lead to greater variability in yield, an undesirable outcome. The long-term impact of increased pollination will be affected by whether or not the crop tends to bear alternately at the floral initiation stage or in the flower-to-fruit conversion stage and by the relative cost of seeds to flowers [7,27]. These aspects of plant development are rarely integrated into models of crop yield but would be straightforward to measure and implement to better align insect conservation and farmer priorities.

We observed signals of numerous environmental shocks in crops at a national scale, but their role as a driver of synchrony in alternate bearing is largely unknown and presents another opportunity for masting theory to inform horticultural understanding. Environmental vetoes—external conditions that prevent seed set—have been well-supported as a driver of synchrony in masting systems [54,55]. As a recent example, Schermer *et al*. [50] studied frost-induced fruit losses in relationship to flower phenology (mean or median data of flowering) in oaks. They concluded that a delay in flowering would lead to a more deterministic pattern of seed production characterized by a lower CV at the population level and a more pronounced lag-1 autocorrelation, which strongly resembles an alternate-bearing pattern (see their fig. 4). By contrast, an advancing (earlier) flower phenology was predicted to increase the stochastic component of interannual variation characteristic of masting. Agricultural studies have offered stronger support for the latter scenario, i.e. advances in flower phenology with climate change, but have not explored the implications of advancing phenology on yield patterns [56,57]. Greater stochasticity in natural systems can be advantageous as a pest control agent (i.e. through predator satiation; [58]); however, farmers are unlikely to experience a net benefit from increased variability given their reliance on a steady income.

Our understanding of mast-seeding would similarly benefit from enhanced integration with agricultural crop data. First, given their direct socio-economic implications, long-term yield datasets are widely available for crop plants and can scale from individual plants to farms to national scales. Our results suggest these data present an underused resource for understanding perennial variability and synchrony. While Gleiser *et al*.'s [42] recent analysis of yield variability across all crops in the FAO data (*n* = 113) found that increasing pollinator dependence was positively associated with yield variability [42], their inclusion of annual plants prevents meaningful inference for perennials. However, they observed greater interannual variability in woody than herbaceous plants, which is consistent with alternate bearing in long-lived perennials [42]. Another difference is that we restricted our analysis to plant species known to be alternate bearing at the individual level. To the best of our knowledge, ours is the first study to use FAO data to assess patterns of yield variability specifically in alternate-bearing plants. Second, the horticultural literature has focused extensively on the genetic and hormonal bases of alternate bearing in an effort to achieve more stable yields [19,25,26]. By contrast, genetic studies in wild-masting trees are scarce but could be valuable for understanding drivers of masting in the wild ([59,60], though see [21] for a notable exception). Finally, the existing variability in farm practices and the potential to manipulate them through space and time (i.e. irrigated versus rainfed; low versus high inputs; monoculture versus agroforestry; self-compatible versus self-incompatible) offer novel opportunities to explore the roles of resource availability, habitat structure, phenology, pollination dynamics and genetics in synchronous perennial plant reproduction.

We observed a national signal of alternate bearing in some insect-pollinated crops (figure 3), despite a stronger tendency toward alternate bearing in wind-pollinated crops. Further work could explore whether crop alternate bearing emerged from a similar pathway across pollination syndromes or as a convergent trait. In wind-pollinated crops, breeding and cultivation may have served to shift the reproductive pattern from stochastic masting toward a relatively more deterministic biennial pattern of reproduction [14]. For insect-pollinated crops, the alternate bearing could be a symptom of agricultural intensification if plants in their native habitat rarely ‘overinvested’ resources in reproduction and instead produced a steady, low number of seeds. Monocultures and high-density planting, for example, can make crops more susceptible to pest outbreaks and more exposed to environmental disasters [61], which would induce synchrony if there were an endogenous resource-driven mechanism involved in alternate bearing. Coffee (*Coffea arabica*) illustrates this well; the plant is native to the shaded understory of Ethiopian rainforest but is now often cultivated in sun monocultures that have been shown to exaggerate alternate bearing and resource tradeoffs (figure 4*b*; [62,63]). Data on the reproductive patterns of wild/ancestral lineages of contemporary perennial crops are scarce [15] but would shed valuable insight on the basis of contemporary alternate bearing.

Unexpectedly, some crop series appeared to display longer-term cycles (3–5 years; figure 4*e*,*f*; electronic supplementary material S7). Such a pattern could arise at farm scales as a result of pruning and subsequent recovery [64], but we would not anticipate these farmer practices to be synchronized at a national scale. Long-term climatic cycles, such as El Niño Southern Oscillation (ENSO) events, could also play a role, particularly in rainfed systems that we may expect to exhibit stronger variability than their irrigated counterparts. ENSO phases have been shown to induce synchrony in masting systems [35,65–67], but knowledge of ENSO effects on crop plants is largely limited to annual crops [68,69]. As a primary source of climate variation in Brazil and Iran, ENSO could be a cause of periodic yield in Brazilian tangerine and Iranian apricot (figure 4*e*,*f*; [70,71]). Climate and pest cycles may also interact to produce complex longer-term cycles in crops [72]. If future work confirms a biological basis of long cycles in some crops at a national scale, it would be profitable to understand the extent to which these longer-term crop fluctuations reflect exogenous forcing (e.g. climate drivers like ENSO) versus endogenous feedbacks (e.g. resource storage and depletion).

In conclusion, we have found that perennial crops frequently exhibit alternate bearing even at a national scale and that this is especially pronounced in wind-pollinated crops. This pattern is remarkable, given the general assumption that management practices have come to outweigh the ecological drivers that would synchronize country-wide production [2,15,25,26]. Our results suggest that historic yield data present a thus far underused resource for further analyses on the mechanisms of reproductive synchrony across time, space and taxa. Future work could explore the intraspecific and intraregional differences in synchrony and the degree of overlap and divergence between patterns in natural and managed systems. We encourage strengthened collaborations between theoretical ecologists, applied ecologists and horticulturalists for the mutual benefits of achieving an enhanced understanding of the mechanisms of synchronous interannual variability and promoting stable yields and farmer livelihoods.

## Data accessibility

We analysed publicly available data collected by the FAO [29]. FAO data subset and R files: https://doi.org/10.6084/m9.figshare.14725182.v1 [73].
